# Letter to the editor: Telemedicine versus conventional care for nonspecific neck pain: a randomized controlled study

**DOI:** 10.55730/1300-0144.6225

**Published:** 2026-02-04

**Authors:** Gülsemin ERTÜRK ÇELİK

**Affiliations:** Division of Physical Medicine and Rehabilitation, Ministry of Youth and Sports, Republic of Türkiye, Ankara, Turkiye

We have carefully reviewed the article by Yılmaz Muluk et al., which compares telemedicine (TM) and conventional care in patients with nonspecific neck pain (NNP). The study presents valuable data on the feasibility of digital health interventions in the field of physical medicine and rehabilitation) [1]. However, we believe that several methodological aspects and certain clinical observations warrant further clarification.

The authors reported that both groups achieved comparable improvements in Visual Analog Scale (VAS) and Neck Disability Index (NDI) scores at the end of three months. However, a notable finding that warrants closer examination is the statistical difference favoring the control group at the 15th-day evaluation (p = 0.038). This early-stage lag observed in the TM group suggests that the initial phase of exercise therapy may require in-person supervision by a rehabilitation specialist to ensure timely biofeedback and accurate correction of movement patterns. In physical therapy, “proprioceptive retraining” frequently depends on manual guidance which may not be adequately replicated through video-based consultations. Moreover, the study’s design did not incorporate advanced remote monitoring, such as AI-based motion analysis, which could potentially have mitigated this early disparity in treatment efficacy.

Furthermore, the study does not adequately account for the potential confounding effect of “digital literacy”. Existing evidence indicates that technological proficiency significantly influence adherence and clinical outcomes in telerehabilitation [2]. In the absence of an assessment of the participants’ digital competence, it remains unclear whether the outcomes observed in the TM group were influenced by the intervention’s efficacy or the patients’ technical limitations.

Another methodological concern relates to the blinding process. Although participant blinding is often impractical in trials of this nature, the paper does not clearly specify whether the outcome assessor was blinded. The absence of assessor blinding may introduce detection bias, particularly in subjective outcome like VAS and NDI, thereby potentially leading to an overestimation of the treatment effect [3].

Lastly, although the reduction in travel time (175.74 min) and distance (38.88 km) represents a clear advantage in terms of patient convenience, the clinical priority ultimately center on the speed and durability of recovery. Given the high recurrence rate of NNP [4], the 3-month follow-up may be insufficient to determine whether patients in the TM group can maintain these improvements as effectively as those who received in-person tactile feedback.

In conclusion, while the study positions TM as a promising modality in rehabilitation practice, we suggest that “hybrid models” or the integration of sensor-based technologies should be further explored to address the early-stage efficacy gap observed in this trial.
